# A new species of *Auyantepuia* González-Sponga, 1978 (Scorpiones, Chactidae) from French Guiana

**DOI:** 10.3897/zookeys.539.6664

**Published:** 2015-11-23

**Authors:** Eric Ythier

**Affiliations:** 1 SynTech Research, 613 route du Bois de Loyse, 71570 La Chapelle de Guinchay, France

**Keywords:** Scorpiones, *Auyantepuia*, new species, French Guiana

## Abstract

A new species of scorpion belonging to the genus *Auyantepuia* González-Sponga, 1978 (family Chactidae Pocock, 1893) is described on the basis of three specimens collected in a rainforest formation located in Saut Sabbat, South of Mana, French Guiana. This is the tenth species of the Guiano-Amazonian genus *Auyantepuia*, and the fifth reported from French Guiana.

## Introduction

In the present paper, a new species of *Auyantepuia* is described from a rainforest formation in French Guiana. The great diversity and endemism in the Guiana region has been previously discussed and evidence from scorpion biogeographic patterns has already been used to support the Guiana region as an important area of endemism (Lourenço 1986, 1991, 2001). The description of the new species raises to ten the number of species belonging to the genus *Auyantepuia* and confirms again the validity of this genus (Lourenço and Qi 2007) and the disrupted and relictual pattern of geographical distribution of the genus, which is confined to the Guiano-Amazon regions, with a strong concentration of the species in the Guayana floristic province (Mori 1991). This also brings further confirmation to the very high levels of endemic species in the Guiana region.

## Methods

Measurements and illustrations were made using a Motic DM143 digital stereo-microscope. Measurements follow Stahnke (1970) and are given in mm. Trichobothrial notations are those developed by Vachon (1974) and the morphological terminology mostly follows Hjelle (1990).

## Taxonomic treatment

### Family Chactidae Pocock, 1893

#### Genus Auyantepuia

Taxon classificationAnimaliaScorpionesChactidae

González-Sponga, 1978

##### Revised diagnosis for the genus.

Scorpions of small size with a total length of 19 to 28 mm. General coloration reddish-brown to dark brown. Tegument smooth overall. Pedipalp chelal fingers very short, with trichobothria *db* and *esb* almost always at the same level; in some species these can be basal to trichobothrium *Et5*. Trichobothrial pattern of type *C*; neobothriotaxic ‘majorante’ (Vachon, 1974). Ventral aspect of metasomal segment V with strong granulations distally, which can form an arc.

##### Composition of the genus *Auyantepuia*

*Auyantepuia scorzai* (Dagert, 1957) (Venezuela)

*Auyantepuia fravalae* Lourenço, 1983 (French Guiana)

*Auyantepuia gaillardi* Lourenço, 1983 (French Guiana)

*Auyantepuia sissomi* Lourenço, 1983 (French Guiana)

*Auyantepuia parvulus* (Pocock, 1893) (Brazil)

*Auyantepuia kelleri* Lourenço, 1997 (French Guiana)

*Auyantepuia mottai* Lourenço & Araujo, 2004 (Brazil)

*Auyantepuia amapaensis* Lourenço & Qi, 2007 (Brazil)

*Auyantepuia surinamensis* Lourenço & Duhem, 2010 (Suriname)

***Auyantepuia laurae* sp. n.** (French Guiana)

#### Auyantepuia laurae sp. n.

Taxon classificationAnimaliaScorpionesChactidae

http://zoobank.org/8CC80D99-F670-43AD-938B-B1508684206E

Figs 1 , 2–4 , 5–7 , 8–9 , 10 , 11 , 12 , Table 1

##### Type material.

One female holotype and two female paratypes. French Guiana, near Saut Sabbat, 50 km south of Mana and 50 km east of Saint-Laurent-du-Maroni, under wood log, I/2015 (E. Ythier & G. Roy). Deposited in the Muséum national d’Histoire naturelle (MNHN), Paris. Comparative material examined: *Auyantepuia fravalae*, 1 male holotype (MNHN-RS-8505) and 1 female allotype (MNHN-RS-8506); *Auyantepuia gaillardi*, 1 male holotype, 1 female allotype and 6 female paratypes (MNHN-RS-3311), 4 female paratypes (MNHN-RS-3307) and 1 male paratype (MNHN-RS-3326); *Auyantepuia sissomi*, 1 female holotype (MNHN-RS-3304) and 1 female paratype (MNHN-RS-3309).

##### Etymology.

The specific name refers to Laura Ythier, for her contribution to the collection of the new species.

##### Diagnosis.

Small scorpions, 27.5 to 28.2 mm in total length. Coloration reddish-brown, with carapace, chelicerae, pedipalps and legs intensely marked with darker spots. Body and appendages weakly granulated or smooth; dorso-internal carina of chela inconspicuous; ventral posterior granulations on metasomal segment V weakly marked. Female pectines with 5-6 to 6-6 teeths; male unknown. Trichobothrial pattern of type C neobothriotaxic ‘majorante’.

##### Description.

based on female holotype and female paratypes.

##### Coloration.

General coloration reddish-brown. Carapace reddish-yellow, intensely marked with brownish variegated spots around the ocular tubercle and on the anterior and posterior edges of the carapace; ocular tubercle darker, almost black. Tergites reddish-brown with confluent reddish-yellow spots, on the sides and the middle of tergites, without forming a longitudinal stripe. Metasomal segments reddish-yellow, marked with variegated brownish spots on lateral and dorsal sides of segments I to V and on ventral side of segments IV and V; ventral side of segments I to III yellowish, without spots; vesicle reddish-yellow with basis of aculeus blackish and tip of aculeus reddish. Chelicerae yellowish, with variegated dark brown spots; fingers reddish-yellow with dark brown spots at their basis, reddish teeth. Pedipalps reddish-brown, with longitudinal dark brown spots. Legs yellowish, intensely marked with brownish spots. Venter and sternites yellowish to reddish-yellow; sternum reddish-yellow with darker spots; genital opercle reddish-yellow; pectines pale yellow.

##### Morphology.

Carapace lustrous and acarinate, with some minute punctations; furrows shallow; anterior edge emarginate. Sternum pentagonal, wider than long. Tergites acarinate, almost smooth and shiny, with only minute granulations on their posterior edges. Pectinal tooth count 5-6 to 6-6, fulcra absent. Sternites smooth and shiny, VII acarinate; spiracles rounded in shape. Only metasomal segments IV and V longer than wide; metasomal tegument almost lustrous, without granulation, and with a few punctations; segment V with spinoid granulation ventrally, weakly marked. Carinae on segments I-V vestigial or absent; only dorso-lateral carinae are weakly marked on segments I to IV. Pedipalp femur with dorsal internal, dorsal external and ventral internal carinae moderately marked; internal face weakly granular; other faces smooth. Patella smooth, with vestigial carinae. Chela weakly granulated, almost smooth, with dorso-internal carina weakly marked. Dentate margins on fixed and movable fingers with 6 rows of granules. Chelicerae with dentition typical of the family Chactidae (Vachon, 1963), and with dense setation ventrally and internally. Trichobothriotaxy of type C; neobothriotaxic ‘majorante’ (Vachon, 1974).

**Figure 1.**
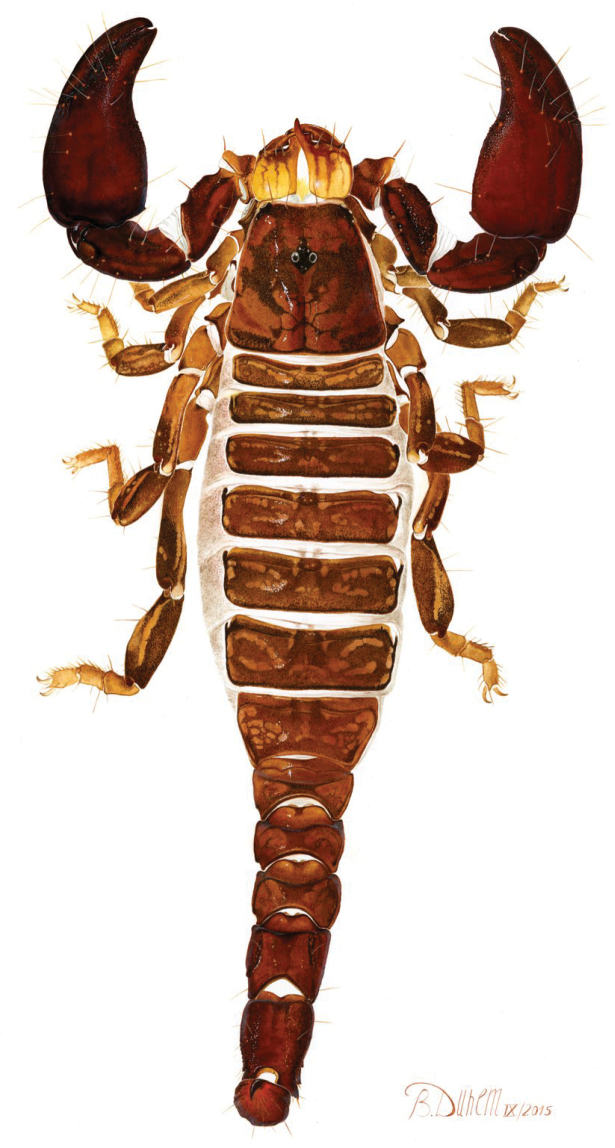
Habitus of *Auyantepuia laurae* sp. n., female holotype.

**Figures 2–4.**
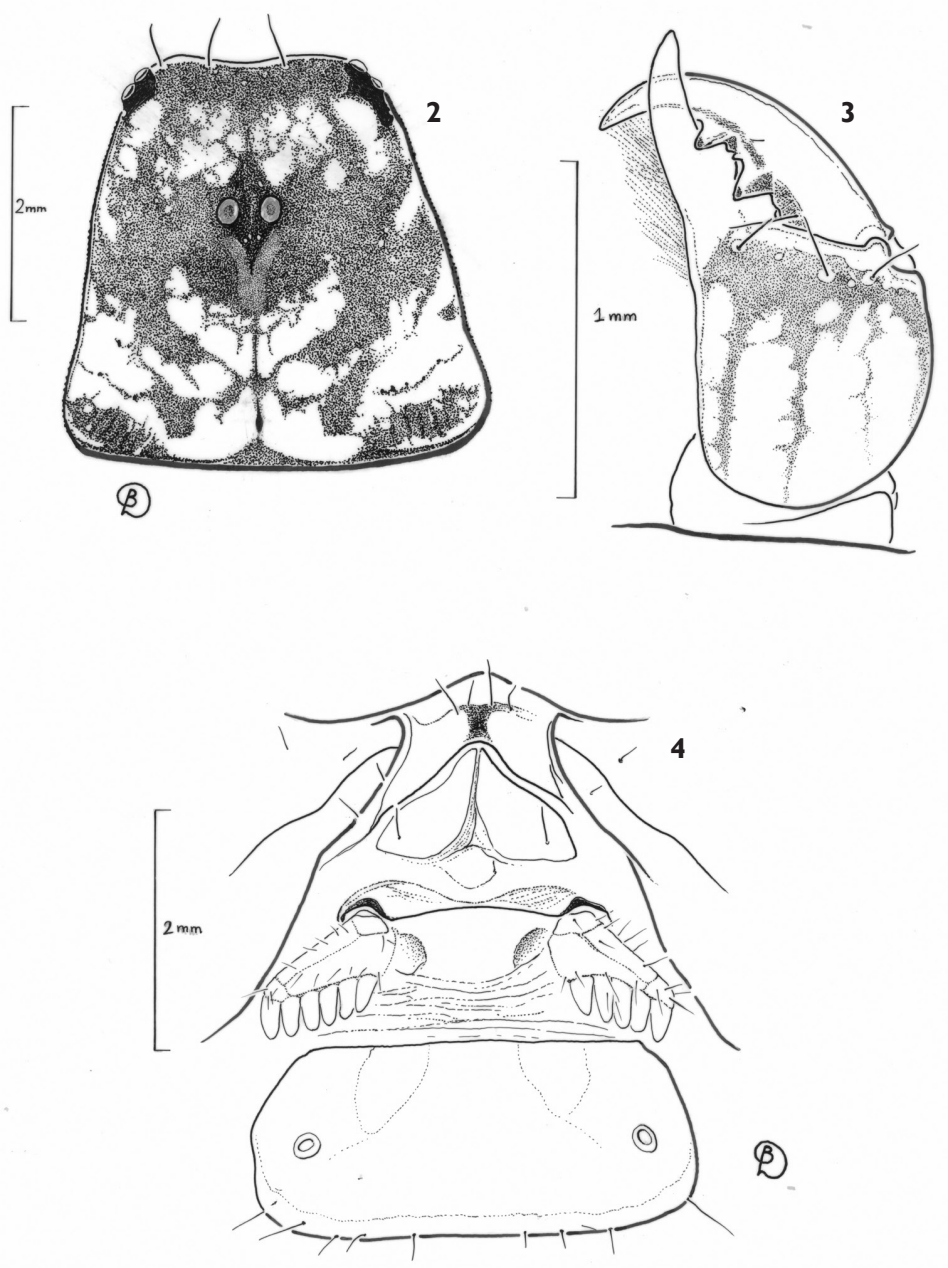
*Auyantepuia laurae* sp. n., female holotype. **2** Carapace, dorsal aspect **3** Right chelicera, dorsal aspect **4** Ventral aspect showing sternum, genital operculum, pectines and sternite III with spiracles.

**Figures 5–7.**
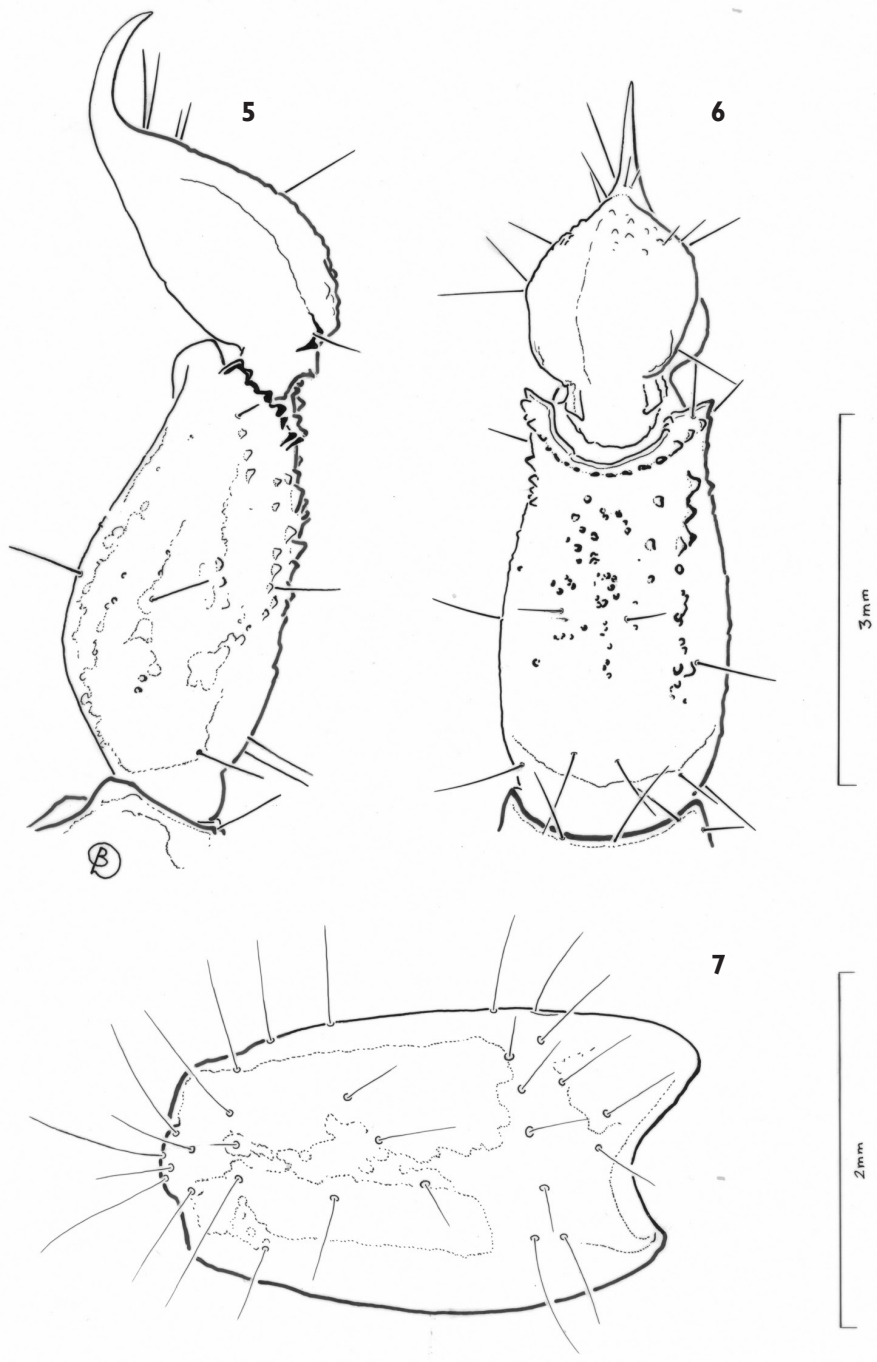
*Auyantepuia laurae* sp. n., female holotype. **5–6** Metasomal segment V and telson, lateral and ventral aspects **7** Patella, external aspect.

**Figures 8–9.**
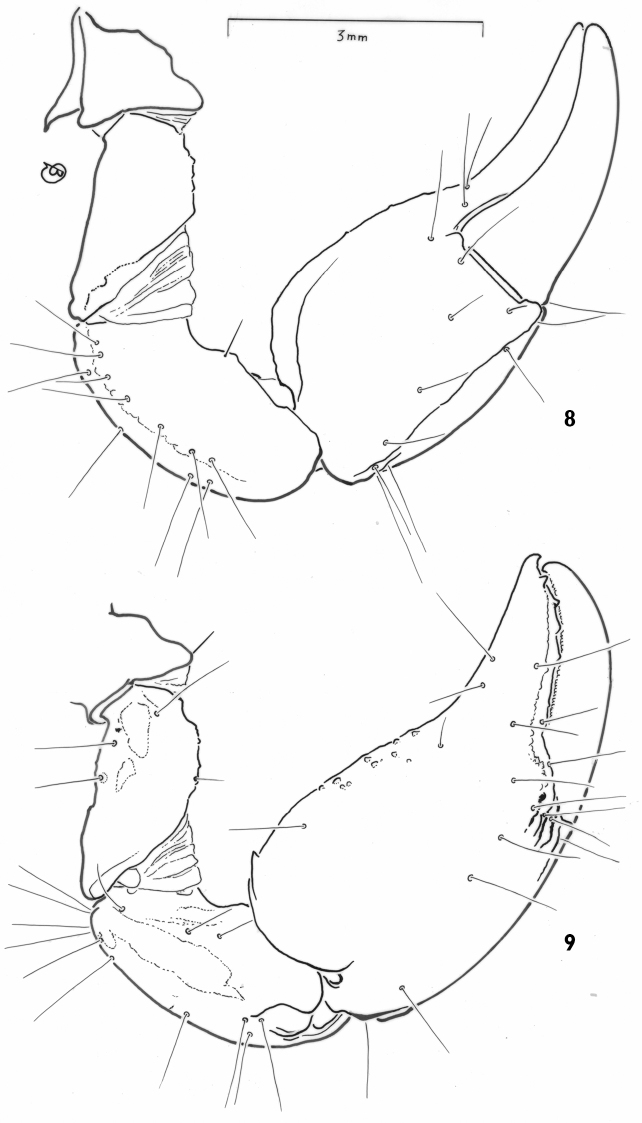
*Auyantepuia laurae* sp. n., female holotype. **8–9** Left (ventral view) and right (dorsal view) pedipalps, showing trichobothrial pattern.

**Figure 10.**
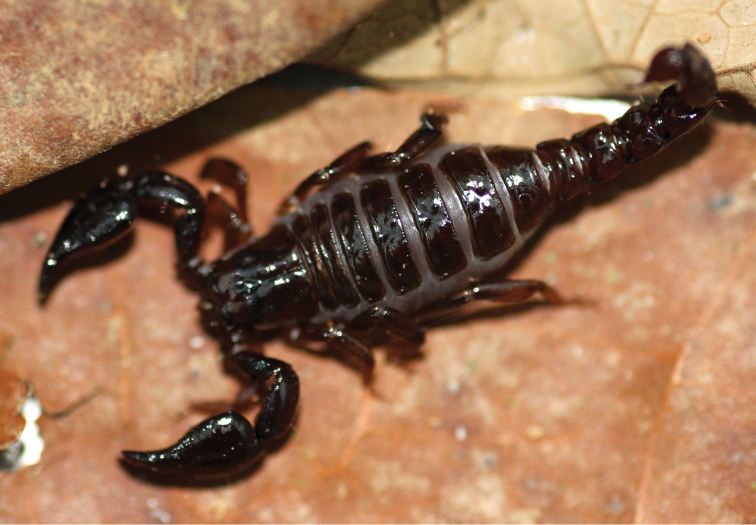
*Auyantepuia laurae* sp. n., female holotype from Saut Sabbat, French Guiana, alive in the field.

**Figure 11.**
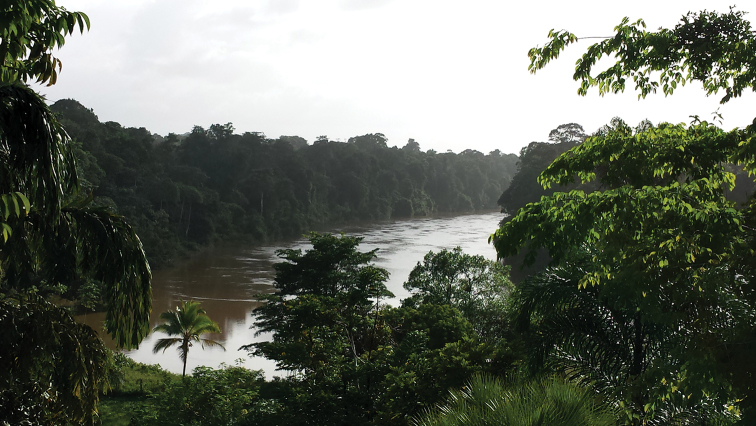
Natural habitat of *Auyantepuia laurae* sp. n. in Saut Sabbat, French Guiana.

**Figure 12.**
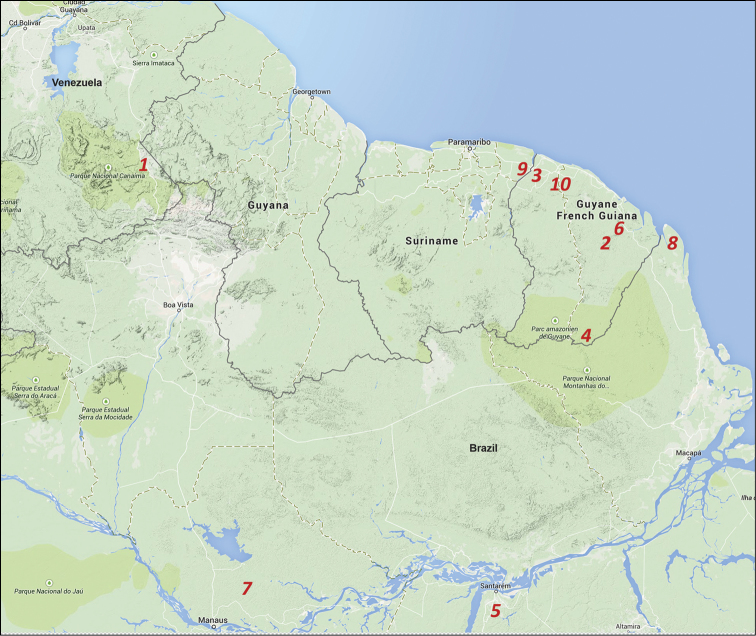
Records of *Auyantepuia* species in Guiano-Amazon regions, tropical South America: *Auyantepuia scorzai* (**1**), *Auyantepuia fravalae* (**2**), *Auyantepuia gaillardi* (**3**), *Auyantepuia sissomi* (**4**), *Auyantepuia parvulus* (**5**), *Auyantepuia kelleri* (**6**), *Auyantepuia mottai* (**7**), *Auyantepuia amapaensis* (**8**), *Auyantepuia surinamensis* (**9**) and *Auyantepuia laurae* sp. n. (**10**).

**Table 1.** Morphometric values (in mm) of *Auyantepuia gaillardi* Lourenço, 1983 (female paratype), *Auyantepuia surinamensis* Lourenço & Duhem, 2010 (female paratype), *Auyantepuia sissomi* Lourenço, 1983 (female holotype), *Auyantepuia fravalae* Lourenço, 1983 (female allotype) and *Auyantepuia laurae* sp. n. (female holotype).

	*Auyantepuia gaillardi* ♀	*Auyantepuia surinamensis* ♀	*Auyantepuia sissomi* ♀	*Auyantepuia fravalae* ♀	*Auyantepuia laurae* sp. n. ♀
Total length	26.9	20.8	26.2	28.6	28.2
Carapace:					
- length	4.1	3.6	3.6	4.3	4.0
- anterior width	2.7	2.3	2.4	2.9	2.1
- posterior width	4.1	3.7	3.6	4.8	4.6
Mesosoma length	7.5	6.0	9.8	9.0	10.5
Metasoma length	15.3	11.2	12.8	15.3	13.7
Metasomal segment I:					
- length	1.8	1.2	1.5	1.7	1.7
- width	2.6	2.2	2.4	2.3	2.6
Metasomal segment II:					
- length	2.1	1.4	1.6	1.8	1.8
- width	2.2	1.9	2.1	2.2	2.1
Metasomal segment III:					
- length	2.5	1.6	1.9	2.0	1.9
- width	2.2	1.8	2.1	2.1	2.0
Metasomal segment IV:					
- length	2.4	1.9	2.1	2.3	2.2
- width	2.0	1.7	1.9	2.0	2.0
Metasomal segment V:					
- length	3.4	2.9	3.0	3.8	3.4
- width	1.9	1.5	1.7	1.8	1.8
- depth	1.6	1.3	1.4	1.6	1.4
Telson:					
- length	3.1	2.2	2.1	2.8	2.7
- width	2.4	1.2	1.4	1.5	1.4
- depth	1.6	0.9	0.9	1.3	0.9
Pedipalp:					
- femur length	2.5	2.1	2.1	2.9	2.4
- femur width	1.2	1.2	1.1	1.3	1.0
- patella length	3.0	2.6	2.7	3.3	2.9
- patella width	1.6	1.3	1.3	1.6	1.4
- chela length	5.8	4.7	5.4	6.7	5.5
- chela width	2.2	1.7	2.0	2.4	2.0
- chela depth	3.1	2.0	2.3	2.9	2.8
Movable finger length	3.0	2.3	2.6	3.4	3.0

##### Relationships.

*Auyantepuia laurae* sp. n. can be distinguished from other species of the genus *Auyantepuia* and, in particular, from the five species described from the Guiana region, by the following features:

- *Auyantepuia gaillardi* Lourenço, 1983 (described from Saint-Laurent-du-Maroni, French Guiana): (i) metasomal segments reddish-yellow, marked with variegated brownish spots on lateral and dorsal sides of segments I to V and on ventral side of segments IV and V; ventral side of segments I to III yellowish, without spots (all segments uniformely reddish in *Auyantepuia gaillardi*), (ii) body, carapace,chelicerae, pedipalps and legs reddish-brown intensely marked with darker spots (uniform coloration without darker spots in *Auyantepuia gaillardi*).

- *Auyantepuia surinamensis* Lourenço & Duhem, 2010 (described from Albina/Moengo, Suriname): (i) metasomal segments reddish-yellow, marked with variegated brownish spots on lateral and dorsal sides of segments I to V and on ventral side of segments IV and V; ventral side of segments I to III yellowish, without spots (all segments reddish uniformely and intensely marked with brownish spots in *Auyantepuia surinamensis*), (ii) size 27.5–28.2 mm (19.0–20.8 mm in *Auyantepuia surinamensis*).

- *Auyantepuia kelleri* Lourenço, 1997 (described from Cacao, French Guiana): (i) metasomal segments reddish-yellow, marked with variegated brownish spots on lateral and dorsal sides of segments I to V and on ventral side of segments IV and V; ventral side of segments I to III yellowish, without spots (all segments uniformely dark reddish in *Auyantepuia kelleri*), (ii) pedipalps intensely marked with dark brown spots (weakly marked in *Auyantepuia kelleri*), (iii) occular tubercle darker, almost black (clear in *Auyantepuia kelleri*).

- *Auyantepuia fravalae* Lourenço, 1983 (described from Saut Pararé, French Guiana): (i) metasomal segments reddish-yellow, marked with variegated brownish spots on lateral and dorsal sides of segments I to V and on ventral side of segments IV and V; ventral side of segments I to III yellowish, without spots (brownish spots on lateral and ventral sides of segments I to V and ventral side of segments I to II well pigmented in *Auyantepuia fravalae*), (ii) pedipalps with chelae weakly granulated, almost smooth (moderately to strongly granulated in *Auyantepuia fravalae*), (iii) female pectines with 5–6 to 6–6 teeth (8–8 in *Auyantepuia fravalae*).

- *Auyantepuia sissomi* Lourenço, 1983 (described from Oyapok, French Guiana): (i) metasomal segments reddish-yellow, marked with variegated brownish spots on lateral and dorsal sides of segments I to V and on ventral side of segments IV and V; ventral side of segments I to III yellowish, without spots (only ventral side of segments I to II *yellowish in Auyantepuia sissomi*), (ii) pedipalps with chelae weakly granulated, almost smooth (moderately to strongly granulated in *Auyantepuia sissomi*), (iii) tergites reddish-brown with confluent reddish-yellow spots, on the sides and the middle of tergites, without forming a longitudinal stripe (yellow spots on the middle of tergites forming a longitudinal stripe dividing the tergites in *Auyantepuia sissomi*), (iv) general coloration reddish-brown (yellowish in *Auyantepuia sissomi*).

### Key to the species of *Auyantepuia* described from the Guiana region

**Table:** 

1	Pedipalps with chelae weakly granulated, almost smooth	**2**
–	Pedipalps with chelae moderately to strongly granulated	**5**
2	Ventral side of metasomal segments I to III yellowish, without spots	***Auyantepuia laurae* sp. n.**
–	Ventral side of all metasomal segments well pigmented, brownish to dark reddish	**3**
3	Body, pedipalps, legs and chelicerae without variegated brownish spots	***Auyantepuia gaillardi***
–	Body, pedipalps, legs and chelicerae marked with variegated brownish spots	**4**
4	Occular tubercle dark, almost black	***Auyantepuia surinamensis***
–	Occular tubercle clear	***Auyantepuia kelleri***
5	Ventral side of metasomal segments I to II yellowish, without spots	***Auyantepuia sissomi***
–	Ventral side of all metasomal segments well pigmented, brownish to dark reddish	***Auyantepuia fravalae***

## Supplementary Material

XML Treatment for Auyantepuia

XML Treatment for Auyantepuia laurae
